# Automatic Detection of the Aortic Annular Plane and Coronary Ostia from Multidetector Computed Tomography

**DOI:** 10.1155/2020/9843275

**Published:** 2020-05-28

**Authors:** Patricio Astudillo, Peter Mortier, Johan Bosmans, Ole De Backer, Peter de Jaegere, Francesco Iannaccone, Matthieu De Beule, Joni Dambre

**Affiliations:** ^1^FEops, Technologiepark- Zwijnaarde 122, Ghent 9052, Belgium; ^2^Department of Electronics and Information Systems, UGent—imec, Technologiepark-Zwijnaarde 126, Ghent 9052, Belgium; ^3^University Hospital Antwerp (UZA), Antwerp, Belgium; ^4^Department of Cardiology, Rigshospitalet University Hospital, Copenhagen, Denmark; ^5^Department of Cardiology, Erasmus MC, Rotterdam, Netherlands

## Abstract

Anatomic landmark detection is crucial during preoperative planning of transcatheter aortic valve implantation (TAVI) to select the proper device size and assess the risk of complications. The detection is currently a time-consuming manual process influenced by the image quality and subject to operator variability. In this work, we propose a novel automatic method to detect the relevant aortic landmarks from MDCT images using deep learning techniques. We trained three convolutional neural networks (CNNs) with 344 multidetector computed tomography (MDCT) acquisitions to detect five anatomical landmarks relevant for TAVI planning: the three basal attachment points of the aortic valve leaflets and the left and right coronary ostia. The detection strategy used these three CNN models to analyse a single MDCT image and yield three segmentation volumes as output. These segmentation volumes were averaged into one final segmentation volume, and the final predicted landmarks were obtained during a postprocessing step. Finally, we constructed the aortic annular plane, defined by the three predicted hinge points, and measured the distances from this plane to the predicted coronary ostia (i.e., coronary height). The methodology was validated on 100 patients. The automatic landmark detection was able to detect all the landmarks and showed high accuracy as the median distance between the ground truth and predictions is lower than the interobserver variations (1.5 mm [1.1–2.1], 2.0 mm [1.3–2.8] with a paired difference −0.5 ± 1.3 mm and *p* value <0.001). Furthermore, a high correlation is observed between predicted and manually measured coronary heights (for both *R*^2^ = 0.8). The image analysis time per patient was below one second. The proposed method is accurate, fast, and reproducible. Embedding this tool based on deep learning in the preoperative planning routine may have an impact in the TAVI environments by reducing the time and cost and improving accuracy.

## 1. Introduction

Aortic stenosis is a progressive valvular heart disease that reduces the motion of the aortic leaflets and valve area [1]. Transcatheter aortic valve implantation (TAVI) has become the preferred treatment for patients with aortic stenosis at high risk for surgical aortic valve replacement (SAVR) [2] and recent clinical data even shows that TAVI is at least as good as SAVR in low-risk patients [3, 4]. During TAVI, a crimped prosthetic valve is positioned in the aortic root and deployed. The calcified native leaflets are crushed against the aortic wall by the expanding metallic device frame and prosthetic leaflets attached to this frame take over the valve's function. The selection of the optimal prosthetic valve size is crucial for the short- and long-term success of the procedure. Incorrect sizing may lead to adverse events where oversizing may cause aortic annulus rupture, coronary obstruction, or conduction abnormalities and undersizing may increase the risk for paravalvular regurgitation or device migration.

Multidetector computed tomography (MDCT) imaging is the gold standard for aortic annulus sizing and TAVI device size selection [5]. The aortic annulus perimeter, area, and diameters are measured at the aortic annular plane (AAP), which is defined by the three basal attachment points of the aortic valve leaflets: the left-coronary cusp (LCC), the noncoronary cusp (NCC), and the right coronary cusp (RCC). The preoperative MDCT images are also used to identify patients at risk for other complications. The distances from the AAP to the coronary ostia (left-coronary ostium (LCO) and the right-coronary ostium (RCO)) are, for example, typically measured to understand the risk of coronary obstruction. This obstruction is a potentially life-threatening complication during which the blood flow to a coronary artery is significantly reduced. Detecting the aforementioned landmarks (LCC, NCC, RCC, LCO, RCO) from MDCT images using the manual method [6] is time-consuming, and both accuracy and reproducibility are strongly dependent on operator experience and the image quality. Considering the rapid expansion of TAVI towards intermediate and low-risk patients, the need for an efficient, automatic, reproducible, and accurate method becomes even more important as optimal sizing and adequate risk assessment are of paramount importance for these patient groups. An automatic method that fulfils the above criteria has the potential to speed up the preoperative planning and to improve device and patient selection, thereby reducing costs and the risk of procedural failure.

In this work, we propose an automated method to extract landmarks for preoperative TAVI planning from MDCT using deep learning techniques. The accuracy and efficiency of the proposed method are assessed on a cohort of 100 patients. The results are compared with an interobserver variability study on the same 100 patients.

## 2. Materials and Methods

### 2.1. MDCT Imaging

This retrospective study used the data of 444 patients collected from multiple centra. The mean age of this cohort was 81 ± 7.3 years. The patient data consisted of electrocardiographic-gated MDCT images which were acquired to support the preoperative phase of a TAVI procedure. Therefore, all MDCT images were contrast-enhanced and contained a certain degree of aortic stenosis. The end-systolic phase was the preferred phase during phase selection [7, 8], however subordinately to the phase with the highest image quality, which led to a cohort consisting of multiple phases. The MDCT images were collected from multiple hospitals which introduced a variety in recording methods and image qualities. Some images showed motion artefacts due to cardiac motion, whereas others displayed metallic artefacts due to the presence of medical devices in the patient. Few images contained regions of noise with no cardiac information. The voxel values represented Hounsfield units (HU), a measure proportional to the degree of X-ray attenuation to discriminate the density of the tissue. The cohort description and scan parameters are given in Table 1. For this retrospective study, formal consent is not required.

### 2.2. Manual Landmark Detection

**Figure 1:**
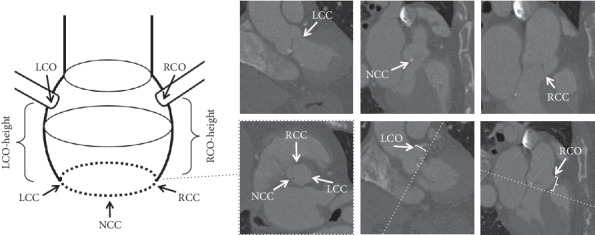
The schematic representation of the anatomy of the aortic root is depicted (left). The top three images identify the three basal attachment points: LCC, NCC, and RCC (from left to right). The bottom three images are (from left to right) the AAP (with the three basal attachment points), the LCO, and the RCO (both with AAP as a dashed line and annotated coronary height).

A trained operator manually detected the five landmarks from the entire cohort with Materialise Mimics Innovation Suite 18 (Mimics, Leuven, Belgium). The LCC, NCC, and RCC were detected following the guidelines [6]; first, the center of the aortic root was detected and used to align the longitudinal axis of the coronal and sagittal plane. Next, the transverse plane was aligned at the level of the valve. The plane was lowered from the aorta towards the ventricle until the basal attachment points of the aortic valve leaflets were visible in this view. Depending on the orientation of the aortic plane, all three basal attachment points needed to disappear at the same time; otherwise, reorientation was required. This reorientation proved to be a difficult task when patients presented with a high calcium load or when the image quality was not sufficient to identify the basal attachment points. The LCO and RCO were visually detected. The AAP was reconstructed from the three basal attachment points, and the coronary heights from the plane to the ostia were measured. These five manually detected landmarks were used as ground truth in this study.  Figure 1 depicts a schematic overview of the landmarks.

A second trained operator redetected the five landmarks blindly for 100 randomly selected patients using the same method mentioned above. The data from the first and second operator were used in the interobserver variability study. The same 100 randomly selected patients were used during the validation of the proposed method. A trained operator analysed the images of these 100 patients and reported that 40% of the images contained movement, 40% contained noise artefacts, 5% contained metallic artefacts due to the presence of medical devices in the patient, and 5% of the images displayed low contrast.

### 2.3. Automatic Landmark Detection

The automatic detection of 3D landmarks in MDCT images can be a difficult task due to the image quality and size variation. Methods to detect landmarks from medical images have already been proposed, and although these methods showed promising results, their coarse accuracy or image modalities did not apply to this problem [9–15]. Therefore, a heatmap detection method similar to [11] is proposed with varying heatmap sizes as an incremental novelty.

This study focussed on automating the manual landmark detection and deriving clinical patient-specific measurements during a postprocessing step. The preprocessing of the ground truth images and manually detected landmarks was necessary in order to prepare the data for further steps of the method.

#### 2.3.1. Preprocessing Step

**Figure 2:**
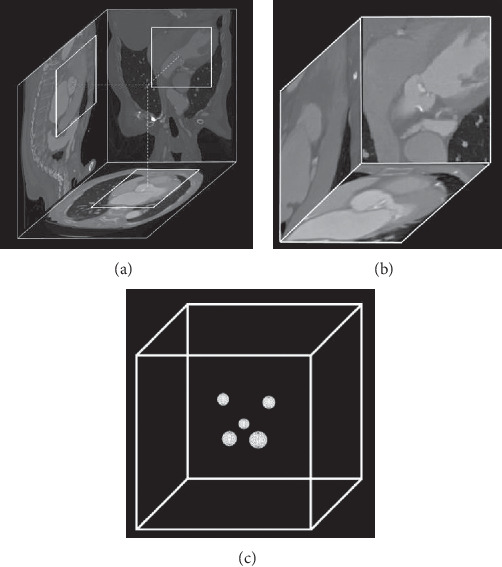
Overview of the MDCT images and the created mask. The extraction of a cube from the full MDCT image (a). An interpolated 128^3^-voxel volume centered around the aortic valve (b). The mask: a zero-valued cube contained five uniquely valued spheres centered around their associated landmark (c).

The volumetric MDCT images were clipped and resampled in order to obtain a homogeneous dataset. Each MDCT image was resampled to an isotropic resolution of 1.0 mm. The original Hounsfield units (HU) were preserved during the resampling process, which was performed with cubic spline interpolation. Next, a 128³-voxel volume, centered around the ground truth landmarks, was extracted from the resampled volumes. The centering was required because the scanned regions differed from patient to patient, ranging from aortic root-specific to entire body scans (Figure 2).

Masks were generated in order to teach the models where to find the target landmarks. A zero-valued 128^3^-cube contained five spheres which were centered at the location of the manually detected landmark in the associated MDCT image. Each sphere was assigned with its own class: LCC = 1, NCC = 2, RCC = 3, LCO 4, RCO = 5 (Figure 2). In order to increase the accuracy, masks with three different sphere radii (3, 5, and 7 mm) were exported.

#### 2.3.2. Architecture

The DenseVNet architecture [16] was used in this study to train the models. This architecture is composed of a downsampling path followed by an upsampling path yielding its *v*-shape. The downsampling path reduces the resolution of the input image to low-resolution representations by using strided convolutions, and the upsampling path increases the output of the downsampling path to the original dimensions by using bilinear upsampling. An input image flows from the downsampling path to the upsampling path followed by the nonlinear softmax activation function, which generates the probability segmentation image.

#### 2.3.3. Training

Three models (N3, N5, and N7) were trained using the training dataset and validated using the validation dataset. One model was trained for each sphere radius (3, 5, and 7 mm). The validation dataset consisted of the same 100 patients that were used for the interobserver variability study, and the training dataset consisted of the remaining 344 patients.

Training details: each model was trained for 860.000 iterations (or 2500 epochs) with the Adam optimiser [17] (with learning rate 1*e*−4) using the Dice coefficient objective function [18]. L2 regularisation with a decay of 1*e*−4 was used in order to prevent overfitting. The network's input size was 64^3^, and a sliding window strategy was applied to the normalised input images. Before a training session, the weights of the model were initialised with random orthogonal matrices [19] with gain equal to $\sqrt{2}$. Finally, ReLU [20] was chosen as the activation function in the convolutional layers.

All hyperparameters were obtained by performing *k*-fold cross-validation on the training dataset (with *k* = 5). The patients were selected at random with a fixed random seed, ensuring the same random order in consecutive cross-validations.

#### 2.3.4. Data Augmentation

A high capacity model and a data-augmentation strategy handled the bias-variance trade-off specific to statistics and machine learning. The trained DenseVNet models yielded a low bias error because it could identify relevant relations between features and targets. A data-augmentation strategy lowered the high variance error caused by the small size of the training dataset. With a low bias and low variance error, the models were able to generalise well beyond the training dataset.

Random image transformations were used in the data-augmentation strategy. The values for random scaling (percentage) and rotating (degrees) were sampled from a uniform distribution ranging between −3 and 3. The random transformations decreased the dependency on the ground truth when centering the 128^3^-voxel MDCT volumes around the five landmarks during the preprocessing step.

### 2.4. Detection

A detection strategy was used to combine the output of the three trained models and use the five predicted landmarks to derive patient-specific anatomical information.

**Figure 3:**
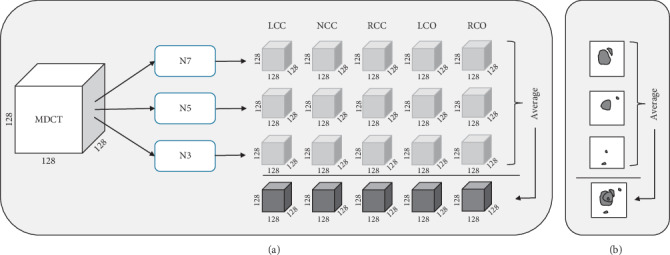
Overview of the detection strategy in 3D (a) and for clarity in 2D (b). The MDCT volume is analysed by the three networks which return five segmentation volumes per network. The output of the three networks is averaged into a probability output volume.

The detection of the five landmarks of a single patient was performed in two steps: a deep learning step and a postprocessing step. During the deep learning step, the volumetric MDCT images were analysed by the three models and the output was combined and normalised into a probability output volume (Figure 3).

**Figure 4:**
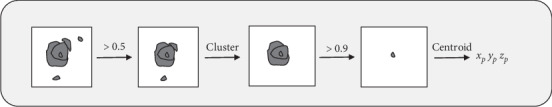
Overview of the postprocessing step depicted in 2D for clarity. From the averaged volume, the points with values higher than 0.5 were clustered. The largest cluster was selected, and from the points with values higher than 0.9, the centroid was computed. This centroid was used as the predicted 3D point.

During the postprocessing step, all voxels with values higher than 0.5 were selected and clustered with hierarchical clustering. The clustering process used the Euclidean distance as the criterion and a threshold of 1.1 mm. From the largest cluster, all points with values higher than 0.9 were used to compute the centroid, which was the final predicted point. This procedure was performed for each of the five landmarks (Figure 4). The output landmarks of the postprocessing step were considered as the final predicted landmarks.

After the detection phase, the aortic annular plane was reconstructed from the LCC, NCC, and RCC and the left and right coronary ostium heights were measured from this plane to the LCO and RCO.

### 2.5. Statistical Analysis

The accuracy of the landmark detection was assessed by measuring the Euclidean distances from the predicted landmarks to the ground truth values. The Shapiro–Wilk test was performed to test for normal distribution, and none of the predicted distributions was normally distributed. All variables were reported as median (lower quartile (LQ)−upper quartile (UQ)). The agreement between manual and the automatic landmark locations was evaluated using the nonparametric signed Wilcoxon test (with a significant *p* value <0.05). Pearson correlation coefficient was computed for the coronary distances to observe their correlation (with excellent correlation *R*^2^ > 0.9). Bland-Altman analysis for the coronary distances was performed. Analyses were performed with SPSS 25.

### 2.6. Implementation

NiftyNet [21], a 3D biomedical deep learning framework that uses Tensorflow [22], was used to train all the models. All the computational work was performed on a multicore computer with Titan X and P6000 GPUs (NVIDIA Corporation, Los Alamitos, CA).

## 3. Results

### 3.1. Detection

The proposed method was validated using the 100 patients also used in the interobserver variability study. By using the same patients for validation and observer variability assessment, it was possible to compare the method with both observers. The predicted landmarks (model) were compared to the ground truth landmarks (observer 1) using the Euclidean distance. The median difference for all landmarks combined was 1.5 mm, which is accurate since the resolution of the MDCT images was 1 mm. The median Euclidean distance for all landmarks between the manually detected landmarks from the first and second observer was 2.0 mm. The distances between model and observer one are lower than the distances between observer one and two, and the overall paired difference of −0.5 ± 1.3 mm is expected because the model was trained with data from observer one (Table 2).

As a final step, the left and right coronary ostium heights were computed from the ground truth and predicted points. There was a small overestimation by the model for the left coronary ostium height (model vs observer one, respectively: median height 16.3 vs 15.8 mm) and a smaller overestimation for the right coronary ostium height (model vs observer, respectively: median height 17.3 vs 17.2 mm) (Table 3).

**Figure 5:**
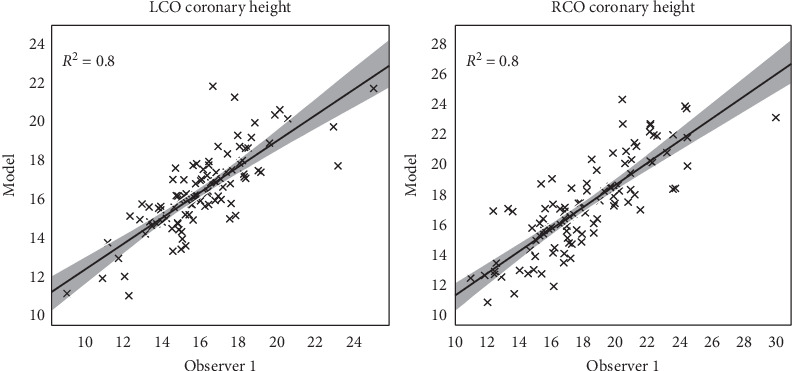
The correlation between the predicted coronary ostium heights and the ground truth from observer 1.

The predicted left and right coronary ostium height correlated well with the ground truth (both with *R*^2^ = 0.8). The left and right coronary height derived from the manually detected landmarks of the second observer correlated well with the ground truth (respectively, *R*^2^ = 0.80 and *R*^2^ = 0.84) (Figure 5).

**Figure 6:**
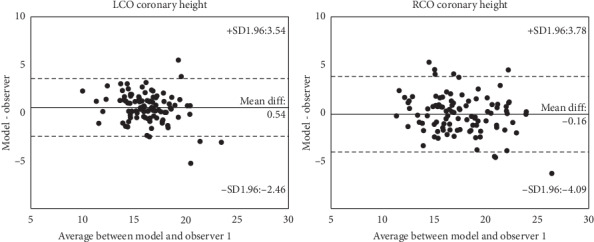
Bland–Altman analysis for left and right coronary ostium height.

Bland–Altman plots for left and right coronary ostium height were created. The left coronary ostium height presented with a mean paired difference of 0.54 mm for observer one, whereas the right coronary ostium height resulted in a mean paired difference of -0.16 mm (Figure 6).

**Figure 7:**
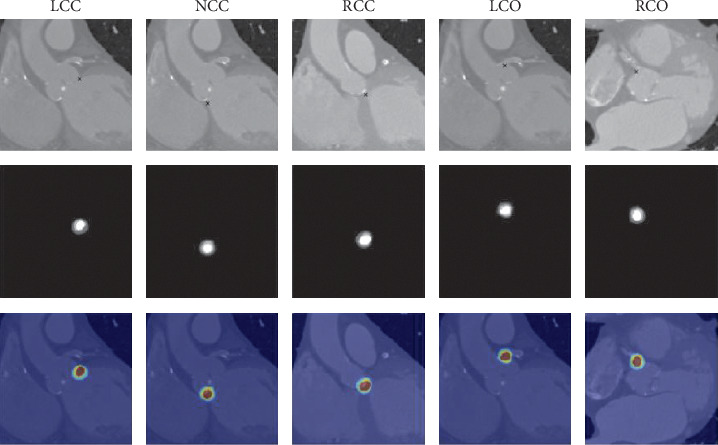
An example of the detected landmarks from an unseen patient. From left to right: LCC, NCC, RCC, LCO, and RCO. From top to bottom: a slice of the MDCT image at the location of the predicted point (annotated), a slice of the averaged final output volume at the location of the predicted point (annotated), and an overlay of the final output volume on the MDCT image at the location of the predicted point (probability 0.0 (blue) to 1.0 (red)).

After the validation of the predicted landmarks and coronary heights, it remains to report the processing time of the manual and automated method. Both observers reported 5 to 10 minutes of analysis time per patient to detect the five landmarks (the time to derive the coronary heights is ignored). The automatic processing time from volumetric MDCT image to the five predicted landmarks and the coronary heights is below 1 second. An example of the predicted output is depicted in Figure 7.

## 4. Discussion

In this work, an automated method is presented to extract landmarks for preoperative TAVI planning from MDCT using deep learning techniques. The validation was performed on 100 patients, and results showed that the five landmarks could be detected efficiently and accurately by combining the results of three models and a postprocessing step. The difference between the manually and automatically identified landmarks was generally smaller compared to differences observed between two operators. These differences indicate that the suggested approach detects these landmarks within acceptable accuracy. Furthermore, it was also illustrated that the method allows determining clinically relevant measurements such as coronary height automatically. The total analysis time from MDCT image data to predicted landmarks is less than 5 seconds, which clearly shows the potential of the proposed method to speed up current preoperative planning workflows.

The literature offers (semi)automatic strategies for landmarks detection in the TAVI field [23–26]. The limitations of the aforementioned works included the following: all studies validated their proposed method with single-center data which leaves the question about the generic property of the method unanswered. Some automatic methods were still operator dependent. Therefore, the robustness of the method cannot be properly assessed, and quality may depend on the experience of the operator. Some studies presented difficulties to adapt to specific pathological conditions, e.g., high calcium load, which is a symptom for requiring a TAVI procedure. The size of the validation cohort was in most studies limited, and the interobserver validation was in few studies absent or not entirely blind.

Our method overcomes almost all the limitations as mentioned above. The method is fully automatic and is insensitive to the amount of calcium load. Although our multicenter patient cohort was relatively small, the number of patients used for training and validation is still higher than the reported literature. We trained our models with 344 patients and validated them with 100 patients where we proved the robustness of the method with a multicentered patient cohort which displayed a good agreement with both observers.

Deep learning methods for landmark detection from medical images can be found in the literature. In Zheng et al. [10], a two-stage classification system for detecting landmarks from head-neck CT scans is proposed, and the reported error is 2.6 ± 5.0 mm. In Payer et al.'s study [11], a method to detect landmarks from 2D CT-scans and 3D magnetic resonance images (MRI) using heatmaps is proposed with a reported error of 1.2 ± 1.3 mm. In Zhang et al.'s study [13], CNNs are used to detect landmarks of the brain from MRI images with a reported error of 3.0 ± 1.6 mm. The same method is applied to detect landmarks of the prostate from CT-scans with a reported error of 3.3 ± 2.5 mm. In Al et al.'s study [15], a colonial walk method is proposed to detect similar aortic anatomical landmarks included in this study. Although the method is fast (12 ms per patient), the overall landmark error for non-TAVI patients is 1.94 ± 0.93 mm and for TAVI patients is 2.74 ± 1.78 mm. In Lalys et al.'s study [27], an automatic segmentation method is proposed. Their results show high precision for the two ostia with distance errors of 1.80 ± 0.74 mm and 1.96 ± 0.87 mm for the LCO and RCO. These errors are slightly higher compared to our results. The study does not include the hinge points of the aortic valve. Finally, in O'Neil et al.'s study [14], a two-pass method for localising 22 anatomical landmarks from head CT scans is proposed. Their method combines a neural network with a landmark Atlas technique. The reported median error of 1.5 mm is equal to our findings (1.5 mm). However, 10.8% of the detected landmarks had an error distance greater than 4.0 mm (computed on 20 scans and a total of 417 landmarks). Our results showed a 0.03% landmark error distance greater than 4.0 mm (computed on 100 scans and a total of 500 landmarks). Only in Payer et al.'s study is the reported error sufficiently fine-grained. Unfortunately, they are obtained from MRI images which have a higher quality than MDCT. Since the reported errors were too coarse-grained or image modalities were not applicable to this problem, a heatmap detection method with varying sizes is proposed.

The potential impact of this work may manifest itself in different frontiers. The method is faster than the state-of-the-art, which may have an impact on reducing operator analysis and errors in a rapidly growing market. Reduced overall TAVI costs may be obtained by embedding the method in software that allows manual corrections (e.g., to correct outliers). This embedding could also yield a continuous learning platform where the coordinates of a new patient, validated by an expert, can be added to the training dataset, thus improving future detections. The coronary ostia heights were derived from the predicted landmarks, which are vital measurements during the preoperative planning of a TAVI procedure [28]. Other clinical parameters, such as the virtual basal ring's minimum and maximum diameter with the circumference, could be derived as well, which could be done by training a CNN model to learn to predict these clinical parameters from images. The same method could be applied for the planning of other cardiovascular interventions, e.g., left atrial appendage occlusion, mitral valve repair/replacement.

Although the presented method has proven to be reliable, there are a few limitations related to the current approach. The maximum outlier for the predicted LCC compared to the first observer was 4.9 mm, which is acceptable when compared to the interobserver difference of 5.8 mm for the same patient. The difference between both observers might indicate a wrong detection by observer one. The maximum outlier of the predicted RCC was 11.5 mm, with an interobserver measurement of 9.4 mm of the same patient. Investigation showed that the images of that patient contained movement, thus making the detection of the landmark difficult for the observer and the algorithm. The predicted measurement lies closer to the measurement of observer two, which may indicate that the models are capable of learning the general location of a landmark and thus contradicting the ground truth. The predicted NCC, LCO, and RCO landmarks presented with maximum outliers smaller than 5 mm. The maximum outliers of the predicted coronary ostium heights were smaller than 5 mm. Although the predictive power has been shown, the outliers indicate that the method is not fail-safe. The final predicted result needs to be validated by an experienced operator, which could benefit the continuous learning method, as described in the previous section. Another limitation of this study is that the method is not applied to patients with anomalies of the coronary arteries [29, 30]. It remains to be tested if the method can be applied to this patient group as well; however, considering the increased risk to sudden cardiac arrest [29], the prevalence of these coronary anomalies in an older patient cohort may be lower. As a final limitation, the MDCT images were of good to excellent quality with a few exceptions of images containing movement or having poor quality. Including poor quality MDCT scans in the training dataset could improve the robustness of the method.

## 5. Conclusion

The proposed method shows that aortic landmark detection from MDCT data is efficient, accurate, and reproducible. Comparison with the interobserver variability has shown the reliability of the strategy and embedding this tool based on deep learning in the preoperative planning routine may have an impact in the TAVI environments by reducing the time and cost and improving accuracy.

## Figures and Tables

**Table 1:** Cohort characteristics and scan parameters.

Age (years)	81 ± 7.3 (56% female, 44% male)
Number of unique contrast agents	11 (90% known)
Number of unique scanners	8 (10% known)
Row (pixels)	512 ± 27.8
Col (pixels)	512 ± 27.8
Depth (slices)	492 ± 306
Pixel space *x* and *y* (mm)	0.5 ± 0.1 and 0.5 ± 0.1
Slice thickness (mm)	0.8 ± 0.3
X-ray tube current (mA)	677 ± 357
Peak kilo voltage output of the x-ray (kV).	107 ± 15
Exposure time (mAs)	589 ± 549
Reconstruction diameter (mm)	271 ± 77

Some parameters were not known or incomplete due to the used anonymisation process. Values are summarised in mean ± std.

**Table 2:** Comparison of the Euclidean distances between the predicted and ground truth target points (*d*_1_) and the target points identified by the two observers (*d*_2_).

Landmark	Model vs ground truth (*d*_1_) (mm)	Observer 1 vs observer 2 (*d*_2_) (mm)	Paired diff. (*d*_1_ − *d*_2_)	*p* value
LCC	1.6 (1.2–2.3)	2.4 (1.4–3.4)	0.8 ± 1.3	<0.001
NCC	1.5 (0.9–2.1)	2.4 (1.4–3.2)	−0.9 ± 1.3	<0.001
RCC	1.6 (1.3–2.2)	2.4 (1.9–3.6)	−0.9 ± 1.4	<0.001
LCO	1.3 (0.9–1.9)	1.4 (1.0–2.2)	−0.1 ± 1.0	0.2
RCO	1.4 (0.9–2.0)	1.4 (1.0–1.8)	0.1 ± 1.0	0.4
All	1.5 (1.1–2.1)	2.0 (1.3–2.8)	−0.5 ± 1.3	<0.001

Paired difference (Paired diff.) is in mm.

**Table 3:** Comparison between the predicted (model) and the ground truth (observer 1) coronary ostium heights.

	Correlation (*R*^2^)	Model (mm)	Observer 1 (mm)	Paired diff. (model − observer 1) (mm)	*p* value
LCO height	0.8	16.3 (15.2–17.7)	15.8 (14.7–17.5)	0.5 ± 1.5	<0.001
RCO height	0.8	17.3 (15.5–19.3)	17.2 (15.1–20.1)	−0.2 ± 2.0	0.4

## Data Availability

The statistical data used to support the findings of this study are available from the corresponding author upon request. The anonymised image data used to support the findings of this study were supplied by FEops N.V. under license and so cannot be made freely available.
